# Hydrogeological and geological partitioning of iron and sulfur cycling bacterial consortia in subsurface coal-based mine waters

**DOI:** 10.1093/femsec/fiaf039

**Published:** 2025-04-09

**Authors:** André Soares, Sara Maria Edwards Rassner, Arwyn Edwards, Gareth Farr, Nia Blackwell, Henrik Sass, Guglielmo Persiani, David Schofield, Andrew C Mitchell

**Affiliations:** Interdisciplinary Centre for Environmental Microbiology (iCEM), Aberystwyth University (AU), Aberystwyth, SY23 3DD, United Kingdom; Department of Life Sciences (DLS), AU, Aberystwyth, SY23 3DD, United Kingdom; Department of Geography and Earth Sciences (DGES), AU, SY23 3DB, Aberystwyth, United Kingdom; Interdisciplinary Centre for Environmental Microbiology (iCEM), Aberystwyth University (AU), Aberystwyth, SY23 3DD, United Kingdom; Department of Life Sciences (DLS), AU, Aberystwyth, SY23 3DD, United Kingdom; Interdisciplinary Centre for Environmental Microbiology (iCEM), Aberystwyth University (AU), Aberystwyth, SY23 3DD, United Kingdom; Department of Life Sciences (DLS), AU, Aberystwyth, SY23 3DD, United Kingdom; British Geological Survey (BGS), Cardiff, CF10 3AT, United Kingdom; Interdisciplinary Centre for Environmental Microbiology (iCEM), Aberystwyth University (AU), Aberystwyth, SY23 3DD, United Kingdom; School of Earth and Ocean Sciences, Cardiff University, Cardiff, CF10 3YE, United Kingdom; Interdisciplinary Centre for Environmental Microbiology (iCEM), Aberystwyth University (AU), Aberystwyth, SY23 3DD, United Kingdom; Department of Geography and Earth Sciences (DGES), AU, SY23 3DB, Aberystwyth, United Kingdom; British Geological Survey, Edinburgh, EH28 8AA, United Kingdom; Interdisciplinary Centre for Environmental Microbiology (iCEM), Aberystwyth University (AU), Aberystwyth, SY23 3DD, United Kingdom; Department of Geography and Earth Sciences (DGES), AU, SY23 3DB, Aberystwyth, United Kingdom

**Keywords:** bacterial community structure, biogeochemistry, Fe-oxidizing bacteria, pyritic coal-based aquifers, S-oxidizing bacteria, subsurface microbial ecology

## Abstract

Pyrite oxidation drives iron and sulfur availability across Earth’s subsurface and is partly microbially mediated. Subsurface microbial communities accelerate this process at circumneutral pH directly by weathering pyritic surfaces and indirectly by causing changes to the surrounding microenvironment, thereby further accelerating pyrite weathering. However, our understanding of community structure dynamics and associated biogeochemistry in Fe- and S-rich lithologies, e.g. pyritic coal, is limited. Here, we present the first comprehensive regional and seasonal genus-level survey of bacterial groundwater communities in a pyritic coal-based aquifer in the South Wales Coalfield (SWC), using 16S rRNA gene amplicon sequencing. Seasonal changes in community structure were limited, suggesting limited influence of surface processes on subsurface communities. Instead, hydrogeologically distinct mine water blocks (MWB) and coal rank largely explained bacterial community structure variation across sites. Fe(II)-oxidizing Betaproteobacteriales genera *Gallionella* and *Sideroxydans* dominated the bacterial communities across nine sites and seven MWBs, while three sites within a single MWB, were dominated by S-oxidizing Epsilonbacteraeota genera *Sulfuricurvum* and *Sulfurovum*. The cooccurrence of pairs of Fe(II)- and S-oxidizing bacterial genera suggests functional redundancy, which coupled with genus-specific morphologies and life strategies, indicates the importance of distinct environmental and ecological niches within the SWC groundwater at seasonal and regional scales.

## Teaser

First spatio-temporal overview of South Wales Coalfield bacterial community structures reveals cooccurrence of Fe- and S-oxidizing bacterial pairs, suggesting hydrogeology and coal rank drive biogeochemistry in this pyritic coal-based aquifer.

## Introduction

**Figure 1.**
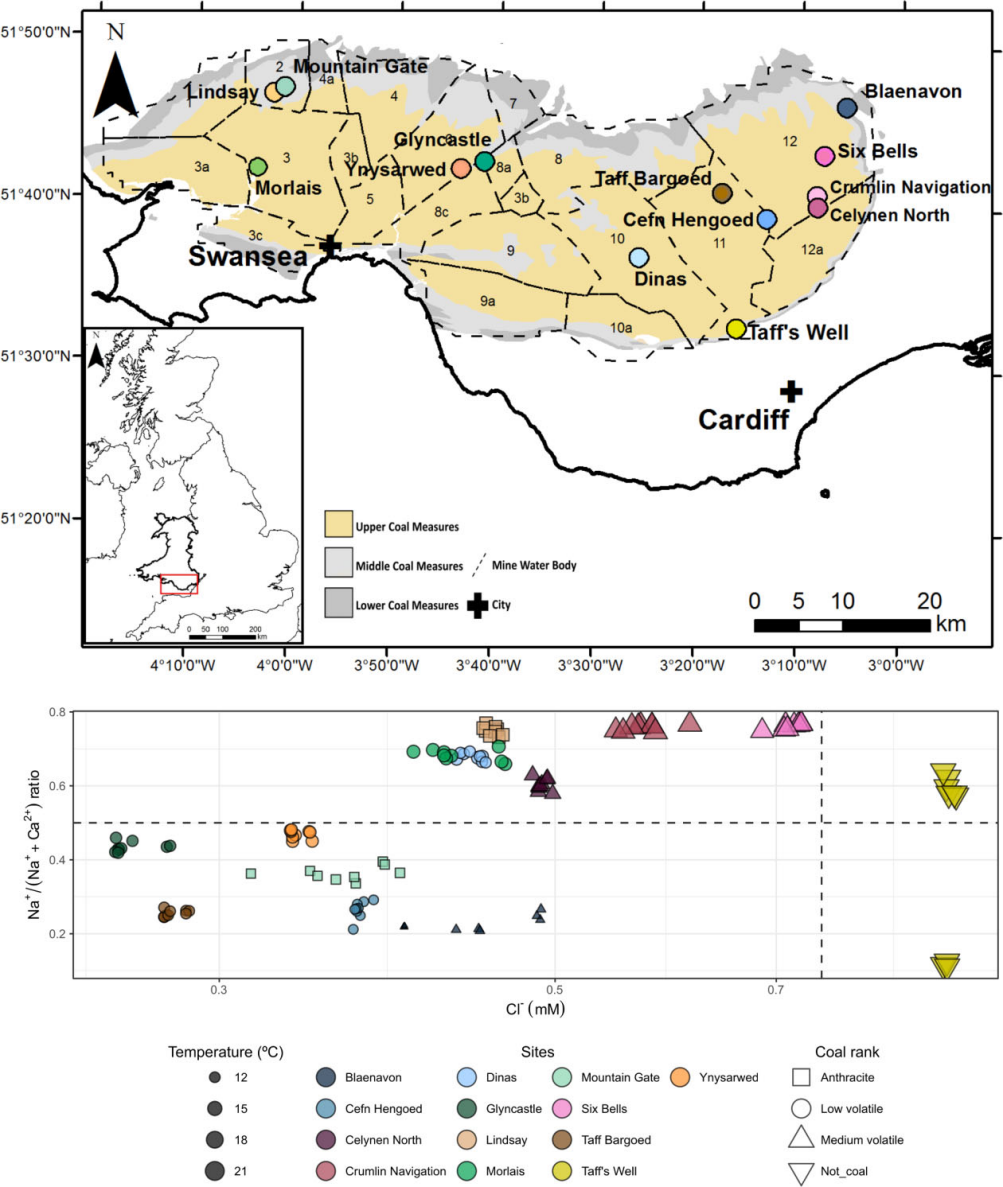
Map of the SWC (top panel), indicating sampling sites (coloured dots) and mine water block divisions according to Lewis et al. (2000b) (©The Coal Authority). Carboniferous Lower, Middle, and Upper Coal Measures indicated by yellow, light gray, and dark gray shading, respectively. Based on British Geological Survey 1:50 000 Bedrock Maps © UKRI. For further information, see Site descriptions and Sampling. Geographically independent groups of samples (bottom panel) were identified based on Na^+^/(Na^+^+Ca^2+^) ratio (indicative of groundwater depth (Robins et al. 2008)) and Cl^−^ concentration (indicative of groundwater age; Freeze and Cherry 1979). The shape of the markers indicates coal rank, whereas their size indicates temperature.

Iron and sulfur are key elements in Earth’s biogeochemical cycles. Weathering of pyrite (FeS_2_), the most abundant iron-sulfide mineral in Earth’s crust, is dependent on both abiotic and microbially mediated processes and is therefore affected by the presence or absence of the bacteria and archaea involved in these processes (Larsson et al. 1990). Depending on its composition, the *in situ* microbial community structure can thus either enhance pyrite weathering or act as a biogeochemical bottleneck in local iron and sulfur elemental cycles (Falkowski et al. 2008). Pyrite oxidation under aerobic conditions releases ferrous iron (Fe^2+^) and sulfate (SO_4_^2−^), with Fe^2+^ being easily oxidized to ferric iron (Fe^3+^) in the presence of oxygen (Moses and Herman 1991). Ferric iron is in turn a strong oxidizer of pyrite, through a reaction resulting in the net release of H^+^, thereby lowering the surrounding pH. While this process occurs abiotically, pyrite weathering can be accelerated by the activity of various bacteria under a range of conditions. A well-known example is one of the most widely studied Fe(II)-oxidizing bacteria, *Acidithiobacillus ferrooxidans*, which is able to accelerate pyrite weathering under aerobic conditions in acidic environments (Hoffmann et al. 1981). However, many more genera have been implied in microbially mediated circumneutral pyrite weathering (Percak‐Dennett et al. 2017, Liu et al. 2021, Napieralski et al. 2022), in a range of environmental settings, including under aerobic (*Thiobacillus, Bradyrhizobium* sp., and *Mesorhizobium* sp.) and anaerobic (*Denitrobacter* and *Sulfurimonas*) conditions. Due to buffering by surrounding geologies, many natural and anthropogenic environments have circumneutral pH and, under these conditions, pyrite oxidation potential increases with O_2_ concentrations (Blöthe and Roden 2009, Percak-Dennett et al. 2017). Although abiotic aerobic pyrite oxidation is possible at circumneutral pH, recent evidence demonstrates the importance of bacterially mediated circumneutral aerobic pyrite oxidation (Napieralski et al. 2022). Chemolithotrophic bacteria, such as genus *Thiobacillus*, oxidize pyrite directly by oxidizing the mineral surface via outer membrane proteins bearing sulfhydryl groups and a persulfide intermediate, leading to the production of sulfate and thin amorphous Fe(III) oxide coatings on the mineral surface (Percak‐Dennett et al. 2017, Napieralski et al. 2022). In pyritic coal-based aquifers, Fe- and S-cycling bacteria often interact syntrophically in multiple ways, e.g. the production of sulfide by sulfate-reducing bacteria resulting in abiotic reduction of Fe(III), thereby further promoting sulfur disproportionation and other, biotically driven, biogeochemical processes (Hansel et al. 2015, Blackwell et al. 2019). However, to date we do not know how communities within pyritic coal-based aquifers are structured over space and time. While the Betaproteobacteriales genus *Gallionella* has recently been identified as a key member of the microbial community in ochreous sediment in the entrance of the Ynysarwed mine adit in the pyrite coal rich South Wales Coalfield (SWC), (Fig. 1), there is currently no large-scale understanding of the community structure dynamics of the microbial communities in the subsurface waters in the SWC (Blackwell et al. 2019).

Here, we present the first comprehensive regional and seasonal survey of genus-level community structure in coalfield groundwater, derived from the flooded mine workings and surrounding aquifers in the SWC, using 16S rRNA gene amplicon sequencing. We (i) demonstrate that microbial communities in the SWC are hydrogeologically compartmentalized by mine water blocks (MWB) throughout the year, regionally separating Fe- and S-cycling consortia, (ii) show that these microbial communities are heavily influenced by the coal rank of the surrounding geology, and (iii) identify codominant pairs of Betaproteobacteriales and Epsilonbacteraeota genera as the putative main Fe- and S-cyclers in the coalfield, indicative of hydrogeological partitioning maintaining separation of partially overlapping niches for pyrite-oxidizing bacteria in subsurface mine waters.

## Materials and methods

### Location, hydrology, geology and mineralogy

The bedrock of the SWC (Fig. 1) is rich in pyrite with a concentration of 2%–4% (wt/wt) (Evans et al. 2006). The SWC covers an area of *ca*. 2200 km^2^ and has been mined since Roman times, with the most significant period of mining occurring from the nineteenth century until the colliery closures in the 1980s (Brown et al. 2002). Structurally the SWC is an east–west trending syncline of Carboniferous Westphalian age strata and can be divided into the Upper, Middle, and Lower Coal Measures (hereafter UCM, MCM, and LCM). The Coal Measures sequence mostly comprises of cyclic sequences of fluvio-deltaic origin, with coarsening upwards series of mudstones, siltstones, and fine-grained sandstones, seatearths coal and numerous marine bands (Robins et al. 2008). The UCM are dominated by thick well-joined feldspathic and micaceous sandstones and some mudstones whilst the MCM and LCM are dominated by mudstones (Robins et al. 2008). Significant fault structures, trending both NE–SW and NW–SE across the SWC, were important for controlling the lateral extent of mining at individual collieries and are reflected in the delineation of a series of hydraulically separate compartments, so-called ‘mine water blocks’ (Fig. 1). MWBs represent flooded collieries that are interconnected and exhibit a continuous gradient of water level across their area (Coal Authority 2018). Conversely, different MWBs are generally hydraulically isolated from each other (Monaghan et al. 2024). Used predominantly as a tool for mine water management by the Mining Remediation Authority (formerly, the Coal Authority), the geology and mineralogy of individual MWBs can be extremely varied, containing strata from the UCM, MCM, and LCM and also workings of coal of various ranks for multiple interconnected collieries. The UCM, consisting mainly of sandstones and mudstones, is known to be more permeable and holds younger, less geochemically evolved mine water (Lewis et al. 2000). Both the MCM and the LCM comprise less permeable mudstones and contain older, more geochemically evolved mine water with reduced flow (Robins and Davies 2016). While siliciclastic mineralogy dominates in the coal measures, the coal measures are sulfur-rich, increasing in sulfur content from west to east as coal rank decreases from anthracite in the west to high volatile coals in the east (Alderton et al. 2004), with pyrite as the primary source, varying in concentrations of 2%–4% (wt/wt) (Evans et al. 2006).

Following the colliery closures in South Wales, dewatering of the mines ceased and the workings were allowed to flood with groundwater. As a result of the increased residence time, the oxidation of pyrite and other iron-sulfide minerals has contributed to the generation of waters highly concentrated in SO_4_^2−^, Fe, and metals (e.g. Zn and Pb), which are transported into the surrounding hydrographical basin (Robins and Davies 2016). Although direct sampling of these underground systems would be prohibitively expensive and logistically difficult, sampling the discharge of bulk waters from flooded mine workings within each discrete MWB offers a potentially attractive system for exploring the interactions between microbes and subsurface hydrochemistry in this pyritic coal-based system. Therefore, we sampled groundwater from discrete MWBs, each characterized by differing hydrogeological regimes, across the SWC and three different coal ranks [anthracite (very low volatile coal), low and medium volatile coals] to characterize the resident bacterial communities using 16S rRNA gene amplicon sequencing in relation to spatial, temporal, and geochemical change.

### Site descriptions and sampling

Groundwater samples were collected from accessible 13 sites (12 gravity-driven or pumped mine water outflows and one thermal spring) in the SWC, UK, in April (12th –14th), August (first, third, and fifth) and December (5th–8th) 2016 (Table S1). The 12 mine water sites represented seven of the 22 hydrogeologically distinct MWBs identified by Lewis et al. (2000). All samples were collected at emerging mine water outflows, as close to the exit as possible in order to reduce interaction with the atmosphere. Moving from west to east through the SWC (Fig. 1), sites *Lindsay* and *Mountain Gate* were affiliated to MWB 2 and lie within the anthracite coal rank with associated very low volatile and sulfide content (George 1970, Alderton et al. 2004, Evans et al. 2006, Chou 2012). *Morlais* is affiliated to block 3, *Ynysarwed* to block 6, *Glyncastle* to block 8, *Dinas is* in block 10, and *Cefn Hengoed* and *Taff Bargoed* in block 11. These lie within the low volatile coal ranks with low sulfide content. MWB 12, holding generally warmer, older and more geochemically evolved groundwater encompassed sites *Six Bells, Crumlin Navigation*, and *Celynen North*, lying within the medium volatile coal ranks with the highest sulfide content of the sites sampled. Despite also being located in MWB 12, mine water at *Blaenavon* is hydrogeochemically very different to the other three sites, as it receives a substantial proportion of its water from the MCM. In contrast, *Taff’s Well* represents an underlying groundwater system draining through Carboniferous limestone and underlying Devonian upper old red sandstone and is considered separate to the overlaying mined layers and thus is not associated with any one MWB. This groundwater is at least 5000 years old and reaches depths of at least 400 m as it follows the base of the synclinal structure of the SWC, recharging in the north before discharging at ∼21°C as a spring in the south of the coalfield (Farr and Bottrell 2013).

For discharge data, average values published by The Mining Remediation Authority (formerly, The Coal Authority) was used (Table 1): Blaenavon (Wyatt and Parker 2024); Cefn Hengoed and Celynen North (Parker and Fox 2024); Dinas (Mallin Martin et al. 2004a); Glyncastle and Ynysarwed (Parker and Wyatt 2024); Lindsay, Morlais and Mountain Gate (Fox and Wyatt 2024); Six Bells (Mallin Martin et al. 2024b); and Taff Bargoed (Mallin Martin et al. 2024c). For Crumlin Navigation, discharge data from Farr et al. (2016) was used and, for Taff’s Well, discharge data from Farr and Bottrell (2013) was used.

**Table 1.** Summary of key features and geochemical variables for each sampling site. Values given as mean (±1 SD). Discharge values are mean values published by The Mining Remediation Authority (formerly The Coal Authority); for references, see the section “Material and methods”: site descriptions and sampling.

Site name	Site type	Block	Coal rank	Discharge (L/s)	Temp (ºC)	pH	Eh (mV)*	EC (µS cm^−1^)	DO (%)	SO_4_^2−^ (mM)	HS^−^ (ug/l)*	Fe^2+^ (mM)
Mt. Gate	FeOB-rich	2	Anthractite	10	11.9 (0.08)	6.26 (0.08)		852 (117)	63.2 (20.0)	1.52 (0.32)		0.0473 (0.00)
Lindsay	FeOB-rich	2	Anthractite	21	15.0 (0.06)	6.81 (0.13)	143 (19.7)	1160 (113)	0.422 (0.89)	2.04 (0.29)	64.3 (7.8)	0.302 (0.01)
Morlais	FeOB-rich	3	Low volatile	125	13.9 (0.07)	6.55 (0.20)	219 (47.5)	1020 (146)	6.53 (10.1)	3.3 0.20)	21.0 (0.0)	0.274 (0.02)
Ynysarwed	FeOB-rich	6	Low volatile	24	13.0 (0.09)	6.25 (0.09)	227 (50.7)	1600 (29.0)	33.1 (1.78)	9.56 (1.58)	31.5 (9.6)	1.45 (0.06)
Glyncastle	FeOB-rich	8	Low volatile	14	12.5 (0.09)	5.95 (0.19)	237	1270 (109)	12.1 (7.11)	7.8 (1.54)	0	0.301 (0.04)
Dinas	FeOB-rich	10	Low volatile	30	12.7 (0.42)	6.64 (0.21)		532 (64.2)	26.1 (31.5)	0.652 (0.04)		0.0868 (0.00)
Cefn Hengoed	FeOB-rich	11	Low volatile	103	11.4 (0.00)	6.64 (0.06)		780 (26.4)	1.56 (2.80)	1.9 (0.08)		0.0506 (0.00)
Taff Bargoed	FeOB-rich	11	Low volatile	100	12.1 (0.12)	7.02 (0.22)		607 (100)	63.0 (15.5)	1.23 (0.12)		0.105 (0.00)
Blaenavon	FeOB-rich	12	Medium volatile	15	10.2 (2.64)	6.71 (0.11)	235	576 (101)	90.3 (10.1)	1.84 (2.86)	5	0.105 (0.01)
Celynen North	SOB-rich	12	Medium volatile	47	13.1 (0.08)	6.89 (0.19)	52 (30.6)	1160 (179)	10.8 (1.58)	2.45 (0.21)	4470 (1710)	0.0546 (0.04)
Crumlin Nav.	SOB-rich	12	Medium volatile	10	19.4 (0.45)	7.33 (0.32)	−59.7 (12.9)	2090 (153)	51.4 (11.9)	2.48 (0.19)	23 600 (2050)	0.000628 (0.00)
Six Bells	SOB-rich	12	Medium volatile	40	18.5 (0.13)	6.99 (0.13)	363	2680 (156)	68.9 (6.95)	10.0 (1.04)	23	0.0249 (0.07)
Taff’s Well	FeOB-rich	–	–	1	21.1 (0.13)	7.26 (0.11)	152	514 (7.40)	24.7 (6.97)	0.233 (0.00)	10	0.0266 (0.00)
Min				10	10.2	5.95	−59.7	514	0.422	0.233	5	0.000 628
Max				125	21.1	7.33	363	2680	90.3	10	23 600	1.45
Median				27	13	6.71	185.5	1020	26.1	2.04	27	0.0868
Mean				44.9	14.2	6.72	166	1142	34.8	3.46	3530	0.218

* Measured from 30 samples collected on 1 June 2021, 1 June 2022, 1 July 2022, 1 September 2022, 1 November 2022, and 1 January 2023.

Lindsay *n* = 6, Morlais *n* = 2, Ynysarwed *n* = 6, Glyncastle *n* = 1, Blaenavon *n* = 1, Celynen North *n* = 6, Crumlin Navigation *n* = 6, Six Bells *n* = 1, and Taffs Well *n* = 1.

At each visit, three independent replicate water samples were collected from the mine adit or outflow at 20–40 min intervals. The collection interval at each site was calculated to ensure that a minimum of three times the underwater volume of the outer adit structure had passed between samples. At each replicate sampling event, water temperature, pH, electrical conductivity, and dissolved oxygen (DO) were measured (Orion Star A329 portable meter, ThermoFisher) and water was collected for geochemical and DNA analysis. Paired 45 ml samples of filtered (0.2 µm cellulose nitrate membrane filter, Whatman) water were collected in sterile 50 ml Falcon tubes, for anion analysis (untreated) and cation and trace element analysis (acidified to pH < 2 with 100 µl of 50% (v/v) trace metal grade HNO_3_). Samples for geochemical analysis were refrigerated upon return to the laboratory and stored at +4°C until analysis. Water for the DNA analysis was collected into a 1 l screw cap polypropylene bottle that had been acid-washed and autoclaved prior to use. The bottle was rinsed with three volumes of sample water immediately before the sample was collected. Samples were refrigerated upon return to the laboratory and filtering of 1 l water samples for DNA analysis through 0.2 µm Sterivex^®^ filters (Merck KGaA) began within 12 h. These were then kept at −20°C until DNA extraction.

### DNA extraction and Illumina amplicon sequencing

DNA was extracted using the DNeasy^®^ PowerWater^®^ Kit (QIAGEN), visualized on a 1% agarose gel (5 µl DNA per lane) and quantified using the Qubit^®^ dsDNA high-sensitivity assay kit (Invitrogen; 5 µl DNA per assay) following manufacturers’ recommended protocols. DNA extracts were stored at −80°C until further use.

The V3–V4 region of the bacterial 16S rRNA gene was amplified through polymerase chain reaction (PCR) using the following recipe: 25 µl Accuzyme Mix (2x, Bioline^®^), 10 µl Forward primer (1 µM, 5′-CCTACGGGNGGCWGCAG-3′), 10 µl Reverse Primer (1 µM, 5′- GACTACHVGGGTATCTAATCC-3′) (Klindworth et al. 2013), and 5 µl sample DNA (final volume 50 µl). Samples were run on a thermal cycler (G-Storm GS1, G-Storm Ltd.) using the following program: 95°C for 3 min, 28 cycles of (95°C for 30 s, 55°C for 30 s, and 72°C for 30 s), followed by a final 72°C step for 5 min. Four PCR controls, in which nuclease-free water was added instead of DNA, were included in the run. Amplification success was confirmed by running 5 µl of PCR product on a 1% agarose gel. Amplification products were cleaned-up using Ampure^®^ beads at a 1.8x ratio and clean-up success confirmed by running amplicons on a 1% agarose gel.

A second-stage PCR was used to add Illumina^®^ Nextera XT indices to the cleaned-up amplicons. The recipe for this stage consisted of 12.5 µl Accuzyme Mix (2x), 2.5 µl Nextera XT Index Primer 1 (5′-CAAGCAGAAGACGGCATACGAGAT [i7 adapter] GTCTCGTGGGCTCGG-3′), 2.5 µl Nextera XT Index Primer 2 (5′-AATGATACGGCGACCACCGAGATCTACAC [i5 adapter] TCGTCGGCAGCGTC-3′), 5 µl PCR-grade water, and 2.5 µl cleaned-up amplicon product (final volume 25 µl). Samples were run on a thermal cycler (G-Storm GS1, G-Storm Ltd.) using the following program: 95°C for 3 min, 8 cycles of (95°C for 30 s, 55°C for 30 s, and 72°C for 30 s), followed by a final 72°C step for 5 min. Success of the second stage PCR was confirmed by running the indexed amplicon products on a 1% agarose gel. A final 1.8x Ampure^®^ beads clean-up was followed by visual confirmation on a 1% agarose gel and Qubit^®^ dsDNA high-sensitivity quantification. Finally, cleaned-up indexed amplicons were pooled in equimolar concentrations in a solution of 10 mM Tris–HCl (pH 8) and 0.1% TWEEN^®^ and subjected to 2 × 300 bp paired-end Illumina MiSeq^®^ sequencing at the Aberystwyth University Illumina sequencing facility.

Sequence data are available at the European Nucleotide Archive (ENA) at EMBL-EBI under accession number PRJEB44467.

### Sequence processing and bioinformatics

Paired-end Illumina MiSeq^®^ reads had their primer and adapter sequences removed using *cutadapt* (https://github.com/marcelm/cutadapt), after which they were subjected to an amplicon sequence variant (SV) calling pipeline by means of the *DADA2* R package (https://github.com/benjjneb/dada2; Callahan et al. 2016), following the standard recommended workflow (https://benjjneb.github.io/dada2/bigdata_paired.html). Forward and reverse reads were trimmed to 260 bp and 200 bp, respectively, following visual analysis of Phred scores. One million reads were subsampled from the forward and reverse FASTQ files in order to generate an error model for the run, which, following dereplication and denoising, was used to accurately call SVs with the *dada* algorithm. Called SVs for each sample’s forward and reverse reads were then merged, after taking into account denoised dereplicated reads, to create an SV table (analogous to an operational taxonomic unit (OTU) table). Bimeras (artefact amplicon sequences composed of two different amplicon sequences, usually as a result of polymerase error) were removed using DADA2’s *de novo* bimera removal function and identical sequences that differed in length were removed using the *CollapseNoMismatch* function. Finally, taxonomy was assigned to species level using the SILVA 132 database (https://www.arb-silva.de/documentation/release-132/, (Quast et al. 2012).

SILVA-assigned taxonomy was imported along with the SV and metadata tables as a *phyloseq* (https://joey711.github.io/phyloseq/index.html; McMurdie and Holmes 2013) object within R. The *decontam* R package (https://github.com/benjjneb/decontam; Davis et al. 2018) was applied to identify potential contaminant SVs by means of their prevalence in true controls. The *phyloseq* object containing SV taxonomy and abundance, as well as the associated metadata (Table S1), was used as the basis for subsequent data exploration and analysis.

### Geochemical analysis

Anion (F^−^, Cl^−^, NO_3_^−^, and SO_4_^2−^) concentrations in prefiltered samples were determined using a Dionex DX 100 ion chromatograph (Thermo Scientific, Sunnyvale, CA, USA), with a 3-point calibration (accuracy ± 4%; precision ± 2%.) Major, minor and trace element (for a total of 54 elements ranging from Li to U) concentrations were determined using an Agilent 7700x ICP-MS (Agilent, Santa Clara, CA, USA). All total dissolved Fe assumed as Fe^2+^ as modeled on PHREEQC (Parkhurst and Appelo 1999) based on the circumneutral pH range (pH 5.95–7.33) of our samples. All samples were run in duplicate and using a 3-point external calibration (accuracy ± 6%; precision ± 2%.). All concentration data passing an initial quality filter were converted to millimolar concentrations and included as metadata in the phyloseq object (Table S1).

Due to repeated probe failure, redox potential (Eh) was not recorded at the time of sampling. Supplemental data for dissolved H_2_S and Eh measurements collected from a subset of sites in 2021 (1st June), 2022 (1st June, 1st July, 1st September, and 1st November) and in 2023 (1st January) were obtained after the main sampling effort. The sites included in this second survey were Lindsay (*n* = 6), Morlais (*n* = 2), Ynysarwed (*n* = 6), Glyncastle (*n* = 1), Blaenavon (*n* = 1), Celynen North (*n* = 6), Crumlin Navigation (*n* = 6), Six Bells (*n* = 1), and Taffs Well (*n* = 1) with a total of 30 samples. Dissolved H_2_S and redox (Eh) measurements were taken immediately at the time of sample collection, with redox potential being measured using an Orion Star A329 portable meter (ThermoFisher) with a Hanna oxidation−reduction potential probe (Ag/AgCl electrode), while H_2_S was determined using an Hach portable spectrophotometer using the Methylene Blue method (Hach Method 8131). The measured values were coherent between both datasets for matched parameters (including pH, temperature, and electrical conductivity; data not shown).

### Statistical analyses

Sequence variant richness (number of SVs present), Shannon’s diversity and Simpson’s evenness indices (Heip et al. 1998) were calculated as per *phyloseq*’s implementation of *vegan*’s *diversity* function (Shannon and Weaver 1949, Dixon 2003). Welch’s independent *t*-test (equal variance not assumed) was used to compare SV richness, Shannon’s diversity, and Simpson’s evenness between Epsilonbacteraeota-rich sites (Celynen North, Crumlin Navigation, and Six Bells) and Betaproteobacteriales-rich sites (all other sites) using the *stat_compare_means* function within the *ggpubr* R package (https://rpkgs.datanovia.com/ggpubr/; Welch 1947). Permutational analysis of variance analysis (PERMANOVA) was performed via the *adonis* function within *vegan* using 1000 permutations with replicates as strata. Constrained analysis of principal coordinates (CAP) ordinations were calculated following the generation of a Jensen−Shannon dissimilarity matrix by means of *phyloseq*’s implementation of *vegan*’s *metaMDS* and *capscale*, respectively (Dixon 2003, Fuglede and Topsoe 2004). The correlation between a subset of geochemical variables (Table 1) were tested using Spearman’s rank correlation test using PAST v4.17 (Hammer et al. 2001). All plots were generated using *ggplot2* (https://github.com/tidyverse/ggplot2; Wickham and Chang 2008). Reproducible *Rmarkdown* scripts for all analyses and figures presented in this paper are available here: https://github.com/GeoMicroSoares/sgg_data_analysis.

## Results

High-throughput 16S rRNA gene amplicon profiles were generated for 117 water samples originating from 12 mine water outflows across the SWC and one thermal spring (Fig. 1, top) across three seasons, resulting in 9656 492 trimmed and quality-filtered reads. In total, 69 596 SVs were generated, of which three were deemed to be contaminants due to their prevalence in negative controls and thus removed from the dataset.

Major ion geochemistry (Table 1, Tables S1 and S2) defined four main hydrogeochemically independent groups of sites across the dataset (Fig. 1, bottom). The Cl^−^ concentration is indicative of the age of the groundwater (Freeze and Cherry 1979), while the Na^+^/(Na^+^+Ca^2+^) ratio is indicative of the depth from which it has been drawn (Robins et al. 2008), with Na^+^-type waters [Na^+^/(Na^+^+Ca^2+^) ratio > 0.5] being typical of deeper groundwater that has had more time to evolve geochemically through weathering of more geochemically inert silicate minerals than shallower or younger Ca^2+^-type waters [Na^+^/(Na^+^+Ca^2+^) ratio < 0.5], which reflect weathering of more geochemically reactive carbonate minerals. Taff’s Well, with its Na^+^-type water, a Cl^−^ concentration above 0.75 mM and a water temperature of *ca*. 21°C, had the deepest, oldest and warmest groundwater in the dataset and sits in a group of its own. Half of the sites (Celynen North, Crumlin Navigation, Dinas, Lindsay, Morlais, and Six Bells) had water of the Na^+^-type, indicative of deeper water that has had more time to evolve geochemically. The remaining sites (Blaenavon, Cefn Hengoed, Glyncastle, Mountain Gate, Taff Bargoed, and Ynysarwed) had cooler water of the Ca^2+^-type and relatively low Cl^−^ concentrations (< 0.5 mM), indicative of the mine water coming from shallower depths and having had less time to evolve geochemically. Of the six sites with Na^+^-type water and Cl^−^ concentrations below 0.75 mM, Six Bells and Crumlin Navigation had particularly high water temperatures (>18°C) and Cl^−^ concentrations (0.55–0.73 mM), suggesting that the mine water at these sites came from greater depths and were older than that of the other mine water sites. The geochemistry of the water at Celynen North, with its Na^+^-type water and Cl^−^ concentrations of 0.48–0.50 mM, places it in an intermediate position within the sites with Na^+^-type water, however this is likely reflecting the influence of younger, shallower groundwater being mixed with the older, deeper mine water as it flows towards the surface. This is further supported by the intermediate water temperature (*ca*. 13°C) at this site (Table 1). Further insight into subsurface biogeochemical conditions and variations between the different sites can be gleaned from comparisons between different geochemical variables. Temperature ranged between 10.2°C at Blaenavon and 21.1°C at Taff’s Well, while DO levels varied between 0.4% at Lindsay and 90.3% at Blaenavon. Dissolved Fe ranged between 0.0006 mM at Crumlin Navigation and 1.45 mM at Ynysarwed and Fe(II) exhibited an overall negative correlation with pH (Spearman’s rank correlation coefficient ρ = −0.61, *P* = .028). Sulfate concentrations ranged between 0.233 mM at Taff’s Well and 10 mM at Six Bells and had a strong positive correlation (ρ = 0.91, *P* < .0001) with electrical conductivity, which ranged from 514 µS cm^−1^ at Taff’s Well to 2680 µS cm^−1^ at Six Bells. There were no other significant correlations between the continuous variables included in Table 1. The difference between sites becomes clearer when including data on redox potential and dissolved sulfide (HS^−^) concentrations from a subsequent sampling campaign. For the nine sites included in both datasets, Eh ranged from 363 mV at Six Bells to −59 mV at Crumlin Navigation, with two sites with the lowest Eh (Celyn North (52 mV) and Crumlin Navigation) exhibiting very high HS^−^ concentrations of < 23 600 ug l^−1^, whereas the remaining seven sites, which all had higher Eh (143–363 mV) had low HS^−^ concentrations of 5–63 ug l^−1^ (Table 1).

**Figure 2.**
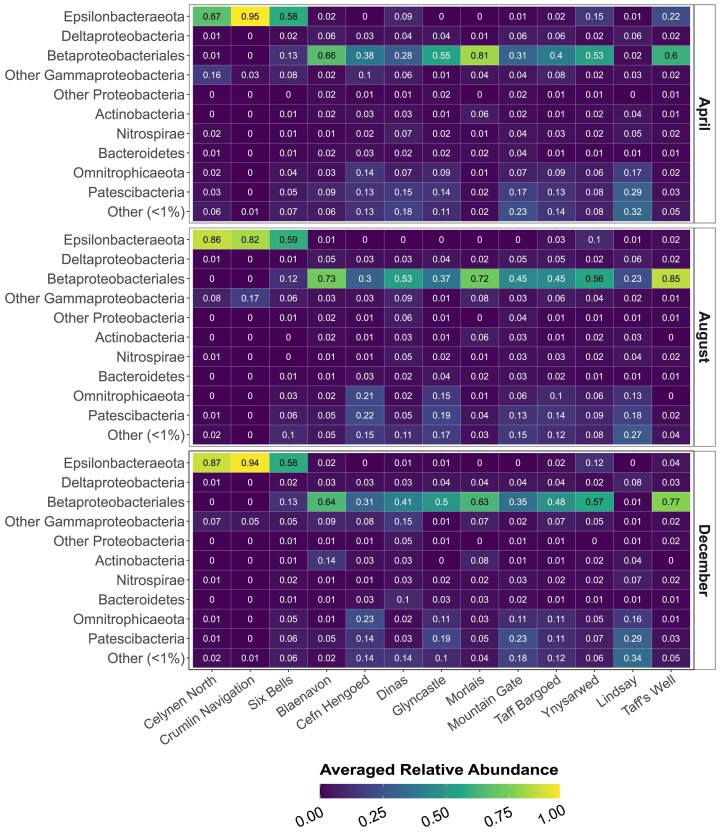
Phylum- and proteobacterial class-level (*y*-axis) relative abundance (averaged from triplicates) across study sites in the SWC (*x*-axis) and month (horizontal panels). SVs representing less than 1% of each phylum were grouped into “Other (<1%)”.

Bacterial communities at all but one (Lindsay) of the sampled sites were dominated by members of either Betaproteobacteriales (Gammaproteobacteria) or Epsilonbacteraeota across all three sampling occasions (Fig. 2) and in total these two taxa represented 57.3% of total number of reads in the dataset. While bacterial communities at the majority of sites were dominated by members of the Betaproteobacteriales, three sites (Celynen North, Crumlin Navigation, and Six Bells) were dominated by members of the Epsilonbacteraeota. At these three sites, all other taxa where only present in very low abundances, with the exception of “Other Gammaproteobacteria” on two occasions [average 16% ± 1% (1 SD) at Celynen North in April and 17% ± 1% at Crumlin Navigation in August].

**Figure 3.**
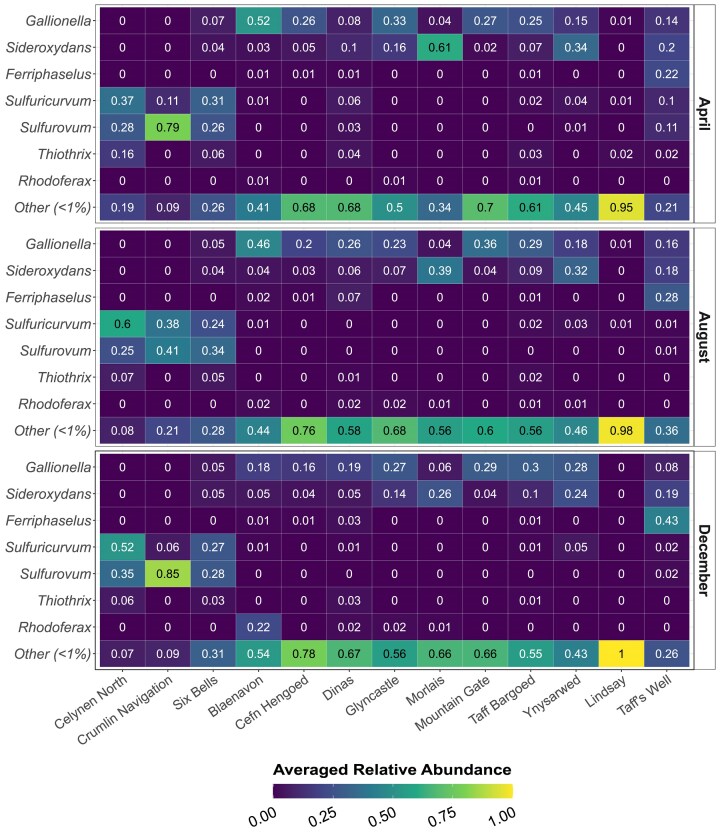
Genus-level (*y*-axis) heatmap of relative abundances (averaged across triplicates) across month (horizontal panels), organized by site (*x*-axis). SVs representing less than 1% of a genus were grouped into one category “Other (<1%).” Each bar corresponds to mean relative abundance of triplicate samples.

For the majority of sites, large parts of their taxonomic profiles could be resolved to genus-level through more fine-grained classification of SVs (Fig. 3). Overall, genus-level resolution was achieved for up to 58.66% of taxonomic profiles in the dataset. Known Fe(II)-oxidizing genera *Gallionella* and *Sideroxydans* (Gallionellales and Gallionellaceae) and S-oxidizing genera *Sulfuricurvum* and *Sulfurovum* (Campylobacterales and Helicobacteraceae) accounted for a total of 54.3% and 93.2% of all the Betaproteobacteriales and Epsilonbacteraeota present in the dataset, respectively.

In Taff’s Well, *Ferriphaselus* (Gallionellales and Gallionellaceae), another known Fe(II)-oxidizing genus, cooccurred with *Gallionella* and *Sideroxydans* across all sampling occasions and even dominated the putative Fe(II)-oxidizing consortium in the December samples (56.4% of all reads for that month and site). Other Betaproteobacteriales genera, such as *Rhodoferax* (Burkholderiales and Comamonadaceae), were also commonly found across the dataset, albeit in smaller average relative abundances, as was genus *Thiothrix* (Thiotrichales and Thiotrichaceae) of Gammaproteobacteria.

In contrast, nondominant taxa were more prominent in the Betaproteobacteriales-rich communities. For instance, Patescibacteria comprised up to 17%±1% (at Mountain Gate) in April, 22%± 0% (at Cefn Hengoed) in August and 23%±1% (at Mountain Gate) in December of the communities in Betaproteobacteriales-rich sites (Fig. 2). In total, 9964 SVs affiliated to 14 Patescibacteria classes were seen across the dataset. Phylum Omnitrophicaeota (previously Candidate Division OP3; Rinke et al. 2013) and Patescibacteria accounted for 37.05% of all reads in the Betaproteobacteriales-rich sample profiles. Interestingly, Patescibacteria and Omnitrophicaeota were the most prominent taxa at Lindsay, with relative abundances of 18%–29% and 13%–17%, respectively (Fig. 2). Actinobacteria were not abundant in the dataset and, apart from a small peak in abundance in Blaenavon during December (10.58±3.3%), they only constituted a small fraction of the total bacterial community across all sites and sampling occasions.

**Figure 4.**
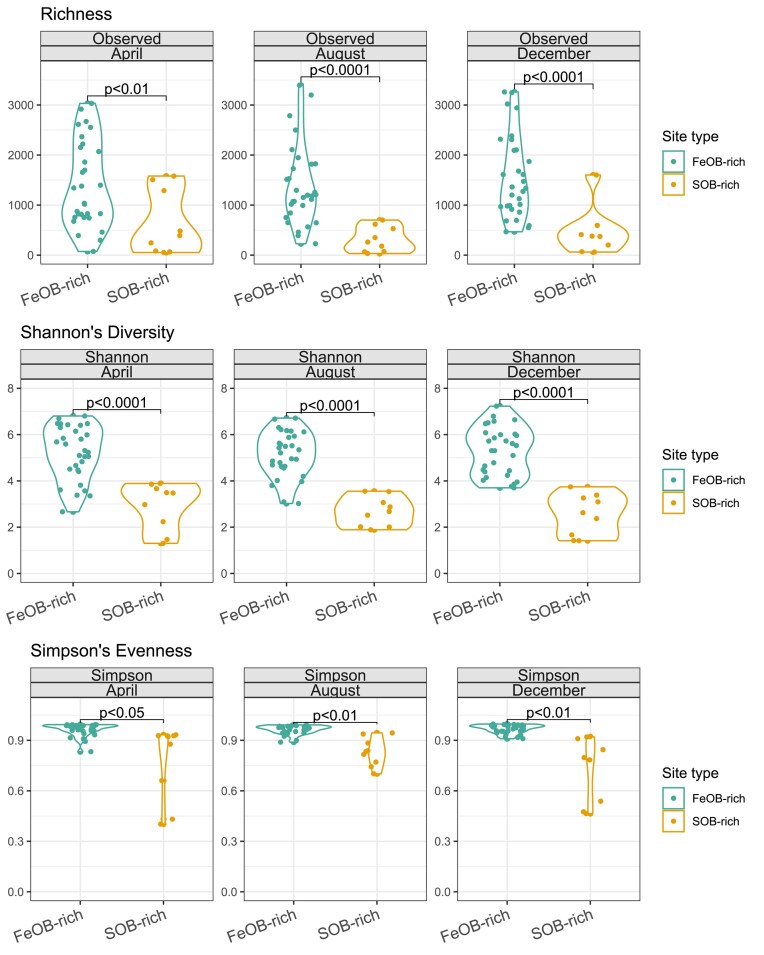
Scatter and violin plots of SV richness (top), Shannon’s diversity (middle) and Simpson’s evenness (bottom) for each month comparing Betaproteobacteriales- and Epsilonbacteraeota-rich sites (labelled FeOB-rich and SOB-rich, respectively). Each dot represents an individual sample. *P*-values indicate significant differences between group means according to Welch’s *t*-test.

Overall, the Betaproteobacteriales-rich communities had significantly higher SV richness (Welch’s *t* tests 2.94, 6.38, 5.04, respectively, *P* < .0001) and Shannon’s diversity (Welch’s *t* tests 5.71, 8.91, 7.81, respectively, *P* < .0001) than the Epsilonbacteraeota-rich communities (Fig. 4). The Simpson’s evenness was also higher in Betaproteobacteriales-rich samples (Welch’s *t* test 2.46, *P* < .05 for April, Welch’s *t* test 4.75 and 3.56 and *P*-values of < .01 for August and December). Seasonality merely explained ∼0.1% (*P* < .0001) of bacterial community structure variation (Table 2) and did not impact SV richness, Shannon’s diversity, or Simpson’s evenness of neither Betaproteobacteriales- nor Epsilonbacteraeota-rich communities (Fig. 4).

**Table 2.** PERMANOVA statistics for included environmental variables and the variation in bacterial community structure explained by each variable. Df = degrees of freedom. Significant *P*-values (*P* < .05) are highlighted in bold.

	*Df*	Sums of squares	*R* ^2^	F Model	*P*-value
MWB	5	7.8309	0.37 756	47.3181	**.000999**
Coal rank	3	6.2882	0.30 318	63.3271	**.000999**
Month	2	0.181	0.00 873	2.7339	**.001998**
Temperature (***°***C)	1	0.5549	0.02 675	16.7652	**.000999**
pH	1	0.3324	0.01 603	10.043	**.000999**
EC (μS cm^−1^)	1	0.2443	0.01 178	7.3813	**.000999**
^56^Fe He	1	0.8615	0.04 153	26.0265	**.000999**
S${\boldsymbol{O}}_4^{2 - }$	1	0.1734	0.00 836	5.2387	**.000999**
Ca^2+^	1	0.112	0.0054	3.3823	**.003996**
DO	1	0.4267	0.02 057	12.8924	**.000999**
Mg*^2+^*	1	0.065	0.00 313	1.9639	.056943
*K* ^+^	1	0.1725	0.00 832	5.211	**.000999**
Na*^2^****^+^***	1	0.0901	0.00 434	2.7215	**.001998**
Cl***^−^***	1	0.2338	0.01 127	7.0629	**.000999**
N${\boldsymbol{O}}_3^ - $	1	0.0305	0.00 147	0.9206	.587413
**^11^** B	1	0.0656	0.00 316	1.9817	**.025974**
Residuals	93	3.0782	0.14 841		
Total	116	20.741	1		

Instead, bacterial community structure variation across sites was largely (68.1%) explained by MWB affiliation (37.8%, PERMANOVA, *F*-statistic = 47.32, *R*^2^ = 0.37756, *P* < .001) and coal rank affiliation (30.3%, *F*-statistic = 63.33, *R*^2^ = 0.30318, *P* < .001, Table 2). The other included variables contributed much less to the total variation, with Fe, temperature, and DO explaining 4.2%, 2.7%, and 2.1% of the variation in microbial community structure, respectively (*P* < .001 for all three). All remaining included variables explained less than 2% of the variation each (Table 2).

**Figure 5.**
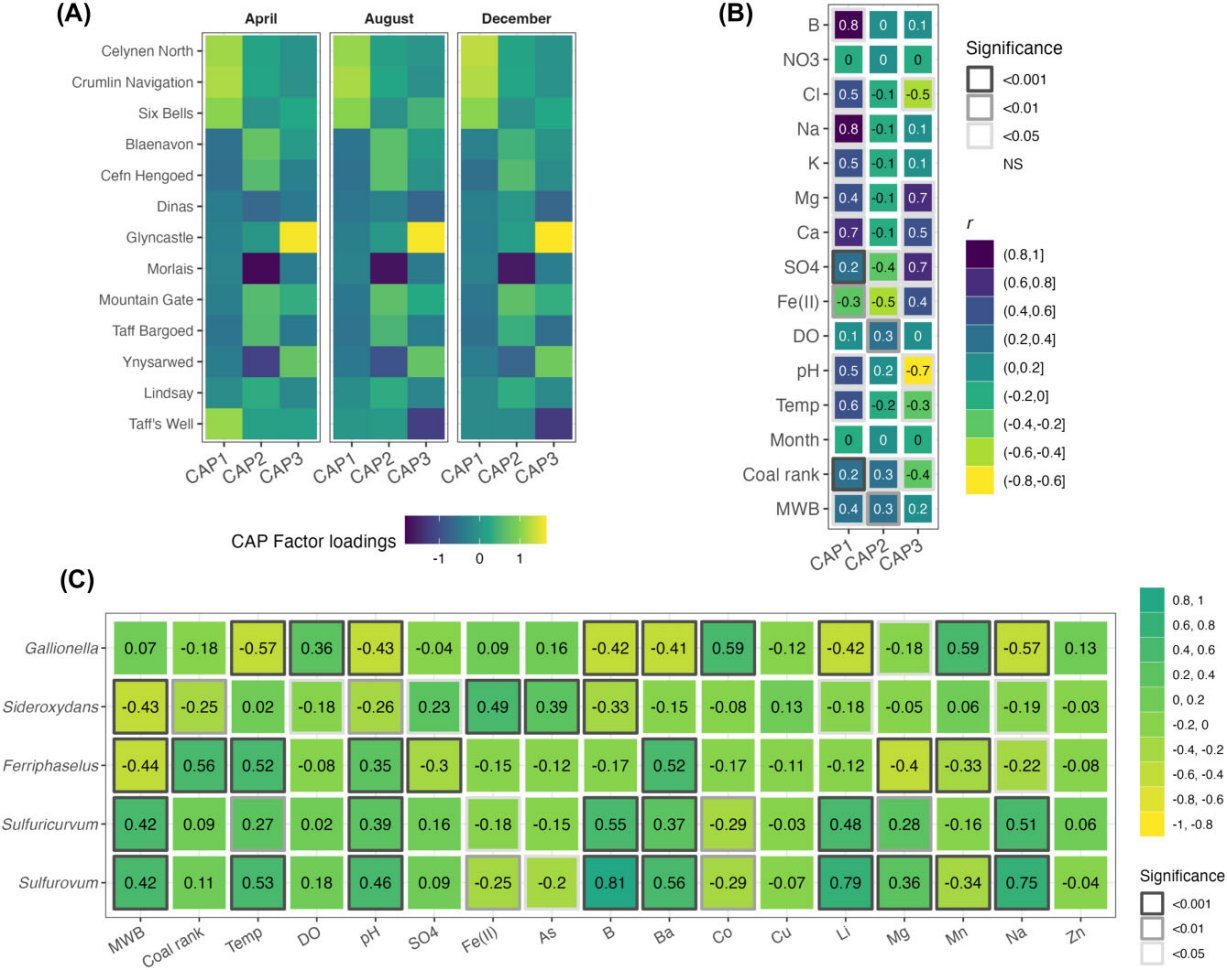
CAP loadings of Jensen–Shannon dissimilarities and normalized environmental variables per site and month (A), and environmental variables (B). Higher loadings are depicted by darker shades, while lower are closer to white. In panel B, transparency of cell borders is proportional to significance. MWB = mine water block, DO = dissolved oxygen, and Temp = water temperature. Heatmap of Pearson correlations between genus-level 16S rRNA gene relative abundances and normalized metadata variables (C). In panel C, significance is indicated by the thickness of each cell contour.

A CAP coordinates explained a total of 44.3% (CAP 1: 22.9%, CAP 2: 11.5%, and CAP 3: 9.9%) of the original Jensen–Shannon dissimilarities. The CAP results highlight, which environmental factors were important in shaping the bacterial community structure of each site (Fig. 5). The first axis delineated the differences between bacterial communities at three of the sites in MWB 12 (Celynen North, Crumlin Navigation, and Six Bells) and at Taff’s Well, on the one hand, and those at all other sites, on the other hand (Fig. 5A). There is less of a clear trend for the second and third CAP axes. For CAP 2, the bacterial communities of Blaenavon, Cefn Hengoed, Mountain Gate, and Taff Bargoed had a higher positive CAP loading factor, whereas Ynysarwed and, in particular, Molais were associated with a negative loading factor. For CAP 3, there was a strong positive correlation with the bacterial communities at Ynysarwed and, in particular, Glyncastle, but also a negative correlation with the bacterial community at Taff’s Well. For the environmental factors, water temperature, pH, and the concentrations of boron (B), Cl^−^, Na^+^, potassium (K^+^), and Ca^2+^ had strong positive correlations with CAP 1 eigenvalues (Pearson’s *r* ≥ 0.5, *P* < .05, Fig. 5B). There were also less strong, positive correlations between CAP 1 eigenvalues and Mg^2+^ and SO_4_^2−^ concentrations (Pearson’s *r* = 0.4 and 0.2, respectively, *P* < .05), as well as a negative correlation between Fe(II) concentration and CAP 1 eigenvalues (Pearson’s *r* = −0.3, *P* < .05). CAP 2 eigenvalues only had significant correlations to three environmental variables, SO_4_^2−^ and Fe(II) concentrations, and DO (Pearson’s *r* = −0.4, −0.5, and 0.2, respectively, *P* < .05). There were more significant correlations between environmental factors and the CAP 3 eigenvalues, including strong positive correlations with Mg^2+^, Ca^2+^, and SO_4_^2−^ (Pearson’s *r* ≥ 0.5, *P* < .05), with some of these correlations being of an opposite sign when compared to CAP 1 correlations (Fig. 5B), i.e. Cl^−^ concentration (Pearson’s *r* = −0.5, *P* < .05), Fe(II) (Pearson’s *r* = 0.4, *P* < .05), pH (Pearson’s *r* = −0.7, *P* < .05) and water temperature (Pearson’s *r* = −0.3, *P* < .05). In comparison, sampling Month did not correlate to CAP 1, CAP 2, or CAP 3 eigenvalues. Coal rank was weakly positively correlated with CAP 1 and CAP 2 eigenvalues, and negatively correlated to CAP 3 (Pearson’s *r* = 0.2, 0.3, and −0.4, respectively, *P* < .05). MWB was positively correlated to CAP 1, CAP 2, and CAP 3 eigenvalues (Pearson’s *r* = 0.4, 0.3, and 0.2, respectively, *P* < .05).

The relative abundances of *Gallionella, Sideroxydans, Sulfuricurvum*, and *Sulfurovum* correlated significantly with pH, B, Li, and Na (Fig. 5C). However, the direction and magnitude of the correlations varied between taxa and each of these four geochemical variables. For example, the correlation with Na was negative for *Gallionella* (Pearson correlation ρ = −0.57, Pearson’s product moment correlation coefficient *P*-value < .001) and *Sideroxydans* (ρ = −0.19, *P* < .01), but positive for *Sulfuricurvum* (ρ = 0.51, *P* < .001) and *Sulfurovum* (ρ = 0.75, *P* < .001). Similarly, the relative abundances of *Gallionella* and *Sideroxydans* were negatively correlated with pH (ρ = −0.43, *P* < .001 and ρ = −0.26, *P* < .01, respectively) and Li (ρ = −0.42, *P* < .001 and ρ = −0.18, *P* < .01, respectively), whereas the relative abundances of *Sulfuricurvum* and *Sulfurovum* were positively correlated with the same variables (pH: ρ = 0.39, *P* < .001 and ρ = 0.46, *P* < .001, respectively; Li: ρ = 0.48, *P* < .001 and ρ = 0.79, *P* < .001, respectively) (Fig. 5C). The relative abundance of *Gallionella* was positively correlated to the two redox metals dissolved manganese (Mn, ρ = 0.59, *P* < .001) and cobalt (Co, ρ = 0.59, *P* < .001) (Fig. 5C). The relative abundance of *Sideroxydans* correlated significantly with Fe (II) (ρ = 0.49, *P* < .001; Fig. 5C) and with arsenic (As, ρ = 0.39, *P* < .001). The relative abundance of *Ferriphaselus* also correlated significantly both positively and negatively with several geochemical variables, however the pattern deviated from both other pairs of genera (Fig. 5C). MWB correlated positively with the relative abundance of *Sulfuricurvum* and *Sulfurovum* (both ρ = 0.42, *P* < .001) and negatively with the relative abundance of *Sideroxydans* and *Ferriphaselus* (ρ = −0.43 and ρ = −0.44, respectively, *P* < .001). There was a positive correlation between the relative abundance of *Ferriphaselus* and coal rank (ρ = 0.56, *P* < .001) and a negative correlation between coal rank and the relative abundance of *Sideroxydans* (ρ = −0.25, *P* < .01).

## Discussion

This work describes the genus-level community structure of groundwater bacteria in a coalfield aquifer with flooded mine workings across 13 sites and three seasons, highlighting stark contrasts between sites despite high within-site stability. Differences in bacterial community structure across sites were best explained by coal rank and MWB affiliation, but not by seasonality. This suggests a fundamental geological control, related to the volatile content as defined by coal rank, and hydrogeological control within each block, as well as confinement from meteoric water inputs (Robins et al. 2008, Robins and Davies 2016, Coal Authority 2018, Monaghan et al. 2024). The high stability of microbial community profiles across site and month indicates a perhaps surprising lack of temporal change across seasons, with the exception of Taff’s Well, where the December samples were distinctly different to those taken from the same site in April and August. This may be due to an inflow of surface or near-surface waters during high flows in December (Farr and Bottrell 2013). MWBs that were characterized by colder, younger, and geochemically less evolved mine water, originating from the UCM and MCM (Fig. 1), and of anthracite or low volatile coal ranks with low sulfide content, presented bacterial communities dominated by Fe(II)-oxidizing Betaproteobacteriales (Fig. 2). In contrast, three sites, Celynen North, Crumlin Navigation, and Six Bells (all located in MWB 12) consistently exhibited community compositions that were distinctly different to those of all the other sites, yet remarkably similar between the three sites, with the communities at all three sites being dominated by S-oxidizing members of Epsilonbacteraeota (Fig. 2). These sites were distinguished by warmer, older, and geochemically more evolved mine water associated with the LCM (Fig. 1; Lewis et al. 2000), as well as of medium volatile coal ranks with higher sulfide content (George 1970, Alderton et al. 2004, Evans et al. 2006, Chou 2012). There were, however, some additional differences independent of MWB and coal rank, e.g. despite Lindsay (MWB 2) being situated only a short distance from the Betaproteobacteriales-rich site Mountain Gate (also MWB 2) and its groundwater being geochemically similar to that of Morlais and Dinas (Fig. 1), the bacterial community at Lindsay had a unique taxonomic profile with stronger contributions from Omnitrophicaeota and Patescibacteria (Fig. 2). Taff’s Well, which drains an underlying system of deeper and older groundwater (Fig. 1 bottom), displayed phylum-level taxonomic similarities with the Betaproteobacteriales-rich sites. However, genus-level taxonomy in this site revealed higher proportions of Fe-oxidizing genus *Ferriphaselus*, followed by *Sideroxydans* and *Gallionella* (Fig. 3), potentially reflecting the influence of the distinct geological and hydrogeological characteristics of this site. Groundwater emerging at Taff’s Well spring is estimated to be *ca*. 5000 years old, however, its path to the surface from depths of *ca*. 400 m in the SWC may cause limited mixing with shallower groundwater (Farr and Bottrell 2013), which may explain the differences seen in both the hydrogeochemical (Fig. 1) and the 16S rRNA gene data (Fig. 3).

Although sites with similar water types (based upon water residence time and source depth, as derived from Cl^−^ concentrations and Na^+^/(Na^+^+Ca^2+^) ratios in Robins et al. (2008)) had very similar bacterial community profiles and despite the observed correlations between environmental factors and CAP eigenvalues, none of the measured water geochemistry variables individually accounted for more than a small proportion of the observed variation in bacterial community structure (Table 2). This included DO, Fe(II), SO_4_^2−^ despite these being key factors in Fe- and S-oxidizing processes. Instead, most of the variation in bacterial community structure between sites is explained by MWB affiliation and coal rank (Table 2) suggesting that the unique hydrogeological characteristics created by the interconnection and continuous hydrological gradient within each block, as well as the volatile and sulfide contents of coal, which dictate coal rank, control the bacterial community structure (Fig. 5).

Almost all of the bacterial communities in the SWC were dominated by either a pair of genera belonging to Betaproteobacteriales or by a pair of genera belonging to Epsilonbacteraeota. The Betaproteobacteriales-rich sites were dominated by the Fe(II)-oxidizers *Gallionella* and *Sideroxydans* (Blöthe and Roden 2009, Fabisch et al. 2013, Kato et al. 2015), whereas the Epsilonbacteraeota-rich sites were dominated by S-oxidizers *Sulfuricurvum* and *Sulfurovum* (Fig. 3) (Hamilton et al. 2015). This apparent functional redundancy, with coexisting but taxonomically distinct bacteria able of encoding the same energy-yielding metabolic functions, contrasts with the expectation that species need to occupy distinct metabolic niches in order to coexist without competition and is suggestive of some underlying mechanism that maintain the cooccurrence of these genera despite niche overlaps. Due to minor differences in metabolic potential or life strategies (e.g. free-living versus attached to mineral surfaces versus biofilm-forming) between the two genera in each pair, small-scale differences in local geochemical conditions (such as heterogeneity in geological and hydrological conditions) may create niches favouring one genus over the other, while still sustaining large populations of both genera. For example, coal seam-associated bacteria have been shown to be dependent on mineral availability and coal surface topology (Probst et al. 2014, Vick et al. 2019a, McLeish et al. 2021). Closely related bacterial taxa isolated from an Australian coal seam have revealed genomic and phenotypic adaptations that allowed their cooccurrence in similar ecological niches (Vick et al. 2019a, b). Likewise, there are many similarities between *Gallionella* and *Sideroxydans*. Both genera comprise microaerophilic, neutrophilic Fe(II)-oxidizers, with similar requirements with regards to growth conditions. Members of *Sideroxydans* are typically able to cope with a wider range of environmental conditions, including being more tolerant of oxygen-depleted conditions than *Gallionella* (Emerson et al. 2013). However, *Gallionella* can maintain active growth at lower temperatures (reported growth ranges of 4°C–30°C and optimal temperature ranges of 17°C–20°C) than *Sideroxydans* (reported growth range 10°C–37°C and optimal temperature of 30°C) (Hallbeck and Pedersen 1990, Emerson et al. 2013, Kato et al. 2014, Khalifa et al. 2018).

Described members of *Gallionella* and *Sideroxydans* are motile, with one polar flagellum and twitching pili, however motility can be a variable trait depending on growth conditions (Emerson et al. 2013, Kato et al. 2015). Whereas *Sideroxydans* has been described as predominantly motile, the type species *Gallionella ferruginea* is only free-living under favourable conditions supporting exponential growth (Emerson et al. 2013). Many (but not all) members of genus *Gallionella* are known to grow long twisted stalks at circumneutral pH and under microaerophilic conditions (Hallbeck and Pedersen 1990, Konhauser et al. 2011, Suzuki et al. 2011). Stalk formation is commonly described as a survival mechanism that allows these Fe(II)-oxidizing bacteria to avoid encrustation by precipitating Fe(III) (oxyhydr)oxides by elevating the cells above the surface on which they are growing. This can also be a strategy to maintain position in the narrow band of a simultaneously microaerobic and stable reduced environment, formed by opposing O_2_ and Fe^2+^ gradients, that is optimal for Fe(II)-oxidizing bacteria (Emerson et al. 2013).

The niche overlap and differences in life strategies between the two Fe(II)-oxidizing genera open up for potential, interesting interactions between these two populations at Betaproteobacteriales-rich sites across the SWC, with the pH- and redox-sensitive stalk-forming capabilities of genus *Gallionella* contrasting against the hypoxia-tolerant and motile nature of genus *Sideroxydans*. Under certain conditions, *G. ferruginea* has been reported capable of oxidizing S^2−^ and S_2_O_3_^2−^, which has a higher energy yield than oxidizing Fe(II), but also causes profuse secretion of extracellular polymeric substances instead of stalk formation (Lütters-Czekalla 1990). Small, fine-scale differences in one or several environmental variables, such as water temperature, DO, Fe^2+^, metal, and metalloid concentrations, which cannot be ascertained from bulk water chemistry, may tip the balance in favour of one of the two genera, as may specific metabolic responses to those conditions (Cooper et al. 2020). However, it might also be the case that there are ample resources available in these flooded mine workings preventing inter- or intraspecies competition from developing. Members of *Ferriphaselus*—a genus of motile, microaerophilic, neutrophilic Fe(II)-oxidizers that was particularly prominent in the Taff’s Well community profiles—are also capable of forming stalks under similar conditions to those that cause stalk formation in *Gallionella*. Fe-oxidizers, including Gallionella and Sideroxydans are commonly found coexisting in iron-rich systems (including wetland root zones; Weiss et al. 2007) and environments affected by acid mine drainage (Mühling et al. 2016, Grettenberger et al. 2020), but work to identify the underlying factors driving these cooccurrence of phylogenetically close Fe-oxidizing bacteria is still ongoing (Emerson et al. 2013). Grettenberger et al. (2020) found that multiple species within a same Fe-oxidizing genus cooccurred in the same ecological niche and associated that with species-level metabolic adaptations. This highlights the potential impact of niche overlap and fluctuations in environmental conditions on the interactions between coexisting species and on the resulting community structure (Kato et al. 2014).

While the measured bulk water geochemistry could not provide sufficient evidence for geochemical control of bacterial community structure, a number of significant correlations were apparent between geochemical variables and genus-level relative abundances (Fig. 5C). Across the board, two groupings were evident, with the pair consisting of *Gallionella* and *Sideroxydans*, and the other pair consisting of *Sulfuricurvum* and *Sulfurovum*. Within each pair, the magnitudes and directions of the correlations were similar, whereas when compared to each other the two pairs showed contrasting trends (Fig. 5C), suggesting opposing geochemical controls on *Gallionella* and *Sideroxydans*, compared to *Sulfuricurvum* and *Sulfurovum*.

The prevalence of *Gallionella* and *Sideroxydans* at the majority of sites in the SWC suggests that these Fe(II)-oxidizers may have crucial impacts on the regional aquatic Fe biogeochemistry. However, only *Sideroxydans* correlated significantly with Fe (II) (Fig. 5C), likely indicating that abiotic processes of Fe^2+^-hydrolysis and -oxidation to solid Fe(III) (oxyhydr)oxides predominate in *Gallionella*-rich sites. In the process of oxidizing dissolved Fe(II) for their metabolic processes, both genera cause the precipitation of Fe(III) (oxyhydr)oxide minerals, a process that may also cause coprecipitation of key dissolved elements, including As (Blackwell et al. 2019). In our sample set, As was positively correlated with the relative abundance of *Sideroxydans*, but not with the relative abundance of *Gallionella* (Fig. 5C), a further indication that *Sideroxydans* may have a role in the formation of Fe(III) (oxyhydr)oxides in the SWC. Interestingly, the relative abundance of *Gallionella* was positively correlated to two redox metals (Mn and Co; Fig. 5C), to which resistance has been previously demonstrated in *Gallionella* (Johnson et al. 2012, Emerson et al. 2013), suggesting that metal resistance could be an additional factor shaping niches in the SWC.

A similar situation is evident for the Epsilonbacteraeota-rich sites, with a codominant pair of S-oxidizing genera consisting of *Sulfurovum* and *Sulfuricurvum*. Members of *Sulfurovum* tend to be nonmotile, facultative anaerobes, with reported growth temperature ranges of 4°C–50°C and reported optimal growth temperatures of 28°C–37°C, whereas *Sulfuricurvum* is composed of motile anaerobes with a narrower growth temperature range of 10°C–35°C and slightly lower reported optimal growth temperature of 25°C (Kodama and Watanabe 2004, Han et al. 2012, Jeon et al. 2017, Xie et al. 2021). While genus *Sulfurovum* has mainly been described as biofilm-forming, *Sulfuricurvum* has been found in diverse planktonic environments, e.g. deep groundwater and lakes (Bomberg et al. 2015, Llorens-Marès et al. 2015). Yet despite their differences, they also have several similarities, e.g. both genera are known to oxidize S and thiosulfate (S_2_O_3_^2−^), as well as tolerating a range of metal-rich conditions (Dahle et al. 2013, Handley et al. 2014, Hamilton et al. 2015). By oxidizing pyrite dissolution-derived sulfur compounds, these sulfur-oxidizing genera are likely important modifiers of the relative proportions of these sulfur compounds within the coalfield. Cultivated facultative anaerobic representatives of coal seam microbiomes have previously provided genomic evidence for less common opportunistic metabolisms that could also benefit *Sulfuricurvum* and *Sulfurovum* in the SWC (Vick et al. 2019b). While dissolved HS^−^ and redox conditions could not be included in statistical analysis because they were not sampled at the same time as other variables, nor at all sites, the high concentrations of HS^−^ and low Eh at the Epsilonbacteraeota-rich sites Crumlin Navigation and Celynen North suggests that highly reducing conditions and high concentrations of reduced sulfur, driven by the high sulfide content of the medium volatile coals, are critical for their dominance. The lack of correlations found between the relative abundances of *Sulfurovum* and *Sulfuricurvum* and SO_4_^2−^ concentrations in the SWC (Fig. 5C) is reflective of the unimportance of oxidized forms of sulfur to these species. The anomalously low HS^−^ and high SO_4_^2−^ at Epsilonbacteraeota-rich site Six Bells likely reflect the unique situation of this site, where water was pumped to the surface from a deeper borehole and then allowed to cascade over a series of high steps. This is likely to have oxygenated the water and increased its Eh, thus driving the late-stage chemical oxidation of HS^−^ to SO_4_^2−^. Overall, the prevalence of these genera in the warmer, more geochemically evolved mine waters of MWB 12, draining coals with higher volatile and sulfide mineral content, suggests that these play a key role in local S biogeochemistry, further supporting the potential for MWB 12 as a S-cycling hotspot in the SWC, due to the rank and sulfide content of the coal.

The geochemical factors that likely control the presence of these genera were not easily captured by the bulk water geochemistry measured. Indeed, the primary geochemical composition of subsurface rocks and sediments in contact with subsurface waters, as well as the formation of secondary Fe and S precipitates, may impart a stronger control on community composition and genus abundance than the measured bulk dissolved geochemical variables, but given the practical limitations of the subsurface sampling of such materials, the sampling of these would not have been feasible. Nevertheless, determining the composition of local bacterial communities could prove a useful tool for improving the delineation of hydrogeological connections in abandoned mines, in addition to traditional hydrogeological and geochemical techniques, especially if combined with establishing the isotopic (δ^2^H and δ^18^O) signature of the mine water to further constrain its sources and trace any contributions from surface waters.

Distinct microbial taxonomic profiles across the SWC were shaped mainly by the hydrological regimes within separate MWBs and the rank and sulfide content of the coal, rather than by bulk water chemistry. The Fe(II)-oxidizing Betaproteobacteriales genera *Gallionella* and *Sideroxydans* codominated most sites in the anthracitic and low volatile coal ranks, while S-oxidizing Campylobacterales genera *Sulfuricurvum* and *Sulfurovum* codominated three sites within MWB 12 and the medium volatile coal ranks. Species-level genomic adaptations are suggested to drive the co-occurrence of Fe- and S-oxidizing bacterial genera pairs. Thus, specific physiological and metabolic characteristics should define similar but differentiated microbial niches in the SWC. The likely distinct characteristics in the biogeochemical roles of planktonic and attached microbial taxa could play an important role in shaping regional Fe- and S- cycling in the SWC and elsewhere, highlighting the importance of genus-level identification of these communities. This work provides the first large-scale, spatio-temporally resolved survey of bacterial groundwater communities in a coal-based aquifer and reveals the importance of hydrogeological units and coal rank in shaping mine water bacterial communities and regional Fe- and S-biogeochemistry.

## Supplementary Material

fiaf039_Supplemental_File
