# Supplementary material for: Hydrogeological and geological partitioning of iron and sulfur cycling bacterial consortia in subsurface coal-based mine waters
Source: FEMS Microbiol Ecol. 2025 Apr 9;101(5):fiaf039. doi: 10.1093/femsec/fiaf039 (PMC12001885; doi:10.1093/femsec/fiaf039)

**Supplementary Information**

**Title:** Hydrogeological and geological partitioning of iron and sulfur cycling bacterial consortia in subsurface coal-based mine waters

Soares, André ^1,2,3a§^, Rassner, Sara Maria Edwards ^1,2§*^, Edwards, Arwyn ^1,2^, Farr, Gareth ^4b^, Blackwell, Nia ^1^, Sass, Henrik ^5^, Guglielmo Persiani^1,3^, Schofield, David ^6^, Mitchell, Andrew C. ^1,3^

1. Interdisciplinary Centre for Environmental Microbiology (iCEM), Aberystwyth University (AU), Aberystwyth, UK
2. Department of Life Sciences (DLS), AU, Aberystwyth, UK
3. Department of Geography & Earth Sciences (DGES), AU, Aberystwyth, UK
4. British Geological Survey (BGS), Cardiff, UK
5. School of Earth and Ocean Sciences, Cardiff University, Cardiff, UK
6. British Geological Survey, Edinburgh, UK

§ - These authors have contributed equally for this work.

* **Corresponding author**: Sara ME Rassner; DLS, Cledwyn Building, Aberystwyth University, Aberystwyth, SY23 3DD, UK; tel.: +4401970-622330; skr@aber.ac.uk

^a^ Current address: Environmental Metagenomics, Research Center One Health Ruhr of the University Alliance Ruhr, Faculty of Chemistry, University of Duisburg-Essen, Essen, Germany

^b^ Current address: Mining Remediation Authority, 200 Lichfield Lane, Mansfield, Nottinghamshire NG18 4RG.

**Table S1** – Simplified table of hydrochemistry metadata associated to this study (see complete table at <https://github.com/GeoMicroSoares/sgg_data_analysis/blob/master/00_Data/SGG_hydro_and_geochemistry_v3_tidy_mM_wctls.csv>). Crumlin Nav. and Mt. Gate are abbreviations for Crumlin Navigation and Mountain Gate, respectively. Block refers to the mine water block. Site type describes sites as dominated by Fe(II)-oxidisers (FeOB) or S-oxidisers (SOB).

| **Site name** | **Month** | **Time point** | **Site type** | **Coal Rank** | **Block** | **Temp (ºC)** | **pH** | **EC (µS cm^-1^)** | **DO (%)** | **SO_4_^2-^ (mM)** | **Fe^2+^ (mM)** | **Outflow** |
| --- | --- | --- | --- | --- | --- | --- | --- | --- | --- | --- | --- | --- |
| Blaenavon | April | 1 | FeOB-rich | Medium volatile | 12 | 10 | 6.73 | 442 | 87.8 | 1.844091 | 0.113201 | Gravity-driven |
| Blaenavon | April | 2 | FeOB-rich | Medium volatile | 12 | 9.9 | 6.67 | 442.5 | 87.8 | 1.842009 | 0.115773 | Gravity-driven |
| Blaenavon | April | 3 | FeOB-rich | Medium volatile | 12 | 9.9 | 6.64 | 442.8 | 87.8 | 1.845652 | 0.113765 | Gravity-driven |
| Blaenavon | August | 1 | FeOB-rich | Medium volatile | 12 | 10.3 | 6.51 | 660.5 | 88.5 | 1.883127 | 0.114951 | Gravity-driven |
| Blaenavon | August | 2 | FeOB-rich | Medium volatile | 12 | 10.3 | 6.79 | 661.4 | 88.7 | 1.893537 | 0.114364 | Gravity-driven |
| Blaenavon | August | 3 | FeOB-rich | Medium volatile | 12 | 10.3 | 6.8 | 661.6 | 88.5 | 1.869595 | 0.11489 | Gravity-driven |
| Blaenavon | December | 1 | FeOB-rich | Medium volatile | 12 | 10.3 | 6.7 | 623.6 | 95.1 | 1.793603 | 0.083411 | Gravity-driven |
| Blaenavon | December | 2 | FeOB-rich | Medium volatile | 12 | 10.2 | 6.68 | 623.9 | 94.5 | 1.818587 | 0.085158 | Gravity-driven |
| Blaenavon | December | 3 | FeOB-rich | Medium volatile | 12 | 10.2 | 6.88 | 624.1 | 94.4 | 1.756649 | 0.085079 | Gravity-driven |
| Cefn Hengoed | April | 1 | FeOB-rich | Low volatile | 11 | 11.4 | 6.67 | 759 | 8.9 | 1.792562 | 0.047359 | Gravity-driven |
| Cefn Hengoed | April | 2 | FeOB-rich | Low volatile | 11 | 11.4 | 6.66 | 740.2 | 0.9 | 1.812341 | 0.045677 | Gravity-driven |
| Cefn Hengoed | April | 3 | FeOB-rich | Low volatile | 11 | 11.4 | 6.65 | 739.2 | 1.5 | 1.809218 | 0.048085 | Gravity-driven |
| Cefn Hengoed | August | 1 | FeOB-rich | Low volatile | 11 | 11.4 | 6.66 | 796.4 | 0.2 | 1.913316 | 0.05374 | Gravity-driven |
| Cefn Hengoed | August | 2 | FeOB-rich | Low volatile | 11 | 11.4 | 6.69 | 796.2 | 0 | 1.906029 | 0.053424 | Gravity-driven |
| Cefn Hengoed | August | 3 | FeOB-rich | Low volatile | 11 | 11.4 | 6.69 | 796.6 | 0 | 1.920603 | 0.053989 | Gravity-driven |
| Cefn Hengoed | December | 1 | FeOB-rich | Low volatile | 11 | 11.4 | 6.65 | 803 | 1.1 | 1.998676 | 0.051602 | Gravity-driven |
| Cefn Hengoed | December | 2 | FeOB-rich | Low volatile | 11 | 11.4 | 6.5 | 797.5 | 0.9 | 1.996594 | 0.050862 | Gravity-driven |
| Cefn Hengoed | December | 3 | FeOB-rich | Low volatile | 11 | 11.4 | 6.59 | 795.7 | 0.5 | 1.976295 | 0.050833 | Gravity-driven |
| Celynen North | April | 1 | SOB-rich | Medium volatile | 12 | 13.1 | 6.84 | 925.1 | 11.8 | 2.33387 | 0.071616 | Pumped |
| Celynen North | April | 2 | SOB-rich | Medium volatile | 12 | 13 | 6.65 | 922.5 | 8.7 | 2.335952 | 0.07178 | Pumped |
| Celynen North | April | 3 | SOB-rich | Medium volatile | 12 | 13 | 6.67 | 922.4 | 9.2 | 2.351567 | 0.071623 | Pumped |
| Celynen North | August | 1 | SOB-rich | Medium volatile | 12 | 13.2 | 7.08 | 1285 | 11.5 | 2.299518 | 0.05102 | Pumped |
| Celynen North | August | 2 | SOB-rich | Medium volatile | 12 | 13.1 | 7.15 | 1280 | 11.6 | 2.305764 | 0.050397 | Pumped |
| Celynen North | August | 3 | SOB-rich | Medium volatile | 12 | 13.1 | 7.04 | 1280 | 11.6 | 2.273494 | 0.050462 | Pumped |
| Celynen North | December | 1 | SOB-rich | Medium volatile | 12 | 13.2 | 6.7 | 1276 | 8.7 | 2.70654 | 0.041664 | Pumped |
| Celynen North | December | 2 | SOB-rich | Medium volatile | 12 | 13.2 | 6.93 | 1284 | 10.6 | 2.707581 | 0.041311 | Pumped |
| Celynen North | December | 3 | SOB-rich | Medium volatile | 12 | 13.1 | 6.97 | 1287 | 13.2 | 2.754425 | 0.041571 | Pumped |
| Crumlin Nav. | April | 1 | SOB-rich | Medium volatile | 12 | 19.3 | 7.09 | 2013 | 29.2 | 2.339075 | 0.000445 | Pumped |
| Crumlin Nav. | April | 2 | SOB-rich | Medium volatile | 12 | 19.3 | 7.34 | 1848 | 45 | 2.382796 | 2.04E-05 | Pumped |
| Crumlin Nav. | April | 3 | SOB-rich | Medium volatile | 12 | 19 | 7.15 | 1839 | 59 | 2.361977 | 0.000291 | Pumped |
| Crumlin Nav. | August | 1 | SOB-rich | Medium volatile | 12 | 19.7 | 7.75 | 2197 | 43.7 | 2.334911 | 9.27E-05 | Pumped |
| Crumlin Nav. | August | 2 | SOB-rich | Medium volatile | 12 | 19.7 | 7.71 | 2197 | 43.7 | 2.471279 | 8.76E-05 | Pumped |
| Crumlin Nav. | August | 3 | SOB-rich | Medium volatile | 12 | 20.1 | 7.72 | 2144 | 61.7 | 2.346362 | 1.34E-05 | Pumped |
| Crumlin Nav. | December | 1 | SOB-rich | Medium volatile | 12 | 19.7 | 6.98 | 2178 | 66.6 | 2.496263 | 0.002126 | Pumped |
| Crumlin Nav. | December | 2 | SOB-rich | Medium volatile | 12 | 18.6 | 7.08 | 2207 | 53.3 | 2.815843 | 0.00147 | Pumped |
| Crumlin Nav. | December | 3 | SOB-rich | Medium volatile | 12 | 19.2 | 7.12 | 2201 | 60.7 | 2.801269 | 0.00111 | Pumped |
| Dinas | April | 1 | FeOB-rich | Low volatile | 10 | 13.5 | 6.62 | 520.6 | 68.7 | 0.701098 | 0.08708 | Pumped |
| Dinas | April | 2 | FeOB-rich | Low volatile | 10 | 13.1 | 6.9 | 422.7 | 67.6 | 0.691417 | 0.087319 | Pumped |
| Dinas | April | 3 | FeOB-rich | Low volatile | 10 | 13 | 6.91 | 421.5 | 67.9 | 0.701931 | 0.087007 | Pumped |
| Dinas | August | 1 | FeOB-rich | Low volatile | 10 | 12.4 | 6.6 | 570.5 | 7.9 | 0.649049 | 0.084219 | Pumped |
| Dinas | August | 2 | FeOB-rich | Low volatile | 10 | 12.4 | 6.71 | 571.8 | 8.2 | 0.649049 | 0.085112 | Pumped |
| Dinas | August | 3 | FeOB-rich | Low volatile | 10 | 12.4 | 6.71 | 571.4 | 8.2 | 0.649049 | 0.086996 | Pumped |
| Dinas | December | 1 | FeOB-rich | Low volatile | 10 | 12.4 | 6.7 | 567.1 | 3.7 | 0.607618 | 0.085101 | Pumped |
| Dinas | December | 2 | FeOB-rich | Low volatile | 10 | 12.4 | 6.37 | 570.6 | 1 | 0.60564 | 0.087977 | Pumped |
| Dinas | December | 3 | FeOB-rich | Low volatile | 10 | 12.4 | 6.28 | 569.3 | 2 | 0.610429 | 0.090173 | Pumped |
| Glyncastle | April | 1 | FeOB-rich | Low volatile | 8 | 12.4 | 5.81 | 1270 | 15 | 6.631024 | 0.282509 | Pumped |
| Glyncastle | April | 2 | FeOB-rich | Low volatile | 8 | 12.4 | 5.78 | 1276 | 14.2 | 6.604999 | 0.279756 | Pumped |
| Glyncastle | April | 3 | FeOB-rich | Low volatile | 8 | 12.4 | 5.81 | 1274 | 14.3 | 6.693482 | 0.279737 | Pumped |
| Glyncastle | August | 1 | FeOB-rich | Low volatile | 8 | 12.6 | 5.87 | 1344 | 3.5 | 6.839219 | 0.355512 | Pumped |
| Glyncastle | August | 2 | FeOB-rich | Low volatile | 8 | 12.6 | 5.87 | 1344 | 3 | 6.958931 | 0.359033 | Pumped |
| Glyncastle | August | 3 | FeOB-rich | Low volatile | 8 | 12.6 | 5.85 | 994.2 | 2.3 | 6.953726 | 0.358011 | Pumped |
| Glyncastle | December | 1 | FeOB-rich | Low volatile | 8 | 12.5 | 6.2 | 1319 | 19 | 9.97256 | 0.26854 | Pumped |
| Glyncastle | December | 2 | FeOB-rich | Low volatile | 8 | 12.5 | 6.2 | 1320 | 18.7 | 9.808606 | 0.262088 | Pumped |
| Glyncastle | December | 3 | FeOB-rich | Low volatile | 8 | 12.5 | 6.19 | 1320 | 18.6 | 9.777377 | 0.266525 | Pumped |
| Lindsay | April | 1 | FeOB-rich | Anthracite | 2 | 15.1 | 6.75 | 1200 | 0.8 | 1.871677 | 0.308231 | Pumped |
| Lindsay | April | 2 | FeOB-rich | Anthracite | 2 | 15 | 6.89 | 1205 | 0 | 1.847734 | 0.283466 | Pumped |
| Lindsay | April | 3 | FeOB-rich | Anthracite | 2 | 15 | 6.9 | 949.3 | 0 | 1.855021 | 0.307824 | Pumped |
| Lindsay | August | 1 | FeOB-rich | Anthracite | 2 | 15 | 6.56 | 1215 | 2.7 | 1.858144 | 0.297924 | Pumped |
| Lindsay | August | 2 | FeOB-rich | Anthracite | 2 | 15 | 6.7 | 1216 | 0 | 1.841488 | 0.30606 | Pumped |
| Lindsay | August | 3 | FeOB-rich | Anthracite | 2 | 15 | 6.72 | 1216 | 0 | 1.824833 | 0.297694 | Pumped |
| Lindsay | December | 1 | FeOB-rich | Anthracite | 2 | 15 | 6.93 | 976.1 | 0.2 | 2.426517 | 0.295991 | Pumped |
| Lindsay | December | 2 | FeOB-rich | Anthracite | 2 | 14.9 | 6.92 | 1236 | 0 | 2.409862 | 0.314014 | Pumped |
| Lindsay | December | 3 | FeOB-rich | Anthracite | 2 | 14.9 | 6.91 | 1234 | 0.1 | 2.453583 | 0.310673 | Pumped |
| Morlais | April | 1 | FeOB-rich | Low volatile | 3 | 13.8 | 6.32 | 827.4 | 28 | 3.054227 | 0.252902 | Pumped |
| Morlais | April | 2 | FeOB-rich | Low volatile | 3 | 13.8 | 6.67 | 828 | 12 | 3.60178 | 0.253719 | Pumped |
| Morlais | April | 3 | FeOB-rich | Low volatile | 3 | 14 | 6.96 | 829.3 | 16.7 | 3.119808 | 0.255256 | Pumped |
| Morlais | August | 1 | FeOB-rich | Low volatile | 3 | 13.9 | 6.31 | 1133 | 0.8 | 3.152078 | 0.289188 | Pumped |
| Morlais | August | 2 | FeOB-rich | Low volatile | 3 | 13.9 | 6.43 | 1134 | 0 | 3.176021 | 0.288892 | Pumped |
| Morlais | August | 3 | FeOB-rich | Low volatile | 3 | 13.9 | 6.45 | 1134 | 0 | 3.166652 | 0.291852 | Pumped |
| Morlais | December | 1 | FeOB-rich | Low volatile | 3 | 13.8 | 6.57 | 1104 | 0.7 | 3.475822 | 0.282813 | Pumped |
| Morlais | December | 2 | FeOB-rich | Low volatile | 3 | 13.8 | 6.62 | 1104 | 0.3 | 3.466453 | 0.277477 | Pumped |
| Morlais | December | 3 | FeOB-rich | Low volatile | 3 | 13.8 | 6.6 | 1104 | 0.3 | 3.443552 | 0.274995 | Pumped |
| Mt. Gate | April | 1 | FeOB-rich | Anthracite | 2 | 12 | 6.25 | 695.4 | 66.2 | 1.408442 | 0.05098 | Pumped |
| Mt. Gate | April | 2 | FeOB-rich | Anthracite | 2 | 11.9 | 6.32 | 696.7 | 29.6 | 1.392827 | 0.053933 | Pumped |
| Mt. Gate | April | 3 | FeOB-rich | Anthracite | 2 | 11.9 | 6.3 | 697.1 | 27.5 | 1.404278 | 0.053254 | Pumped |
| Mt. Gate | August | 1 | FeOB-rich | Anthracite | 2 | 12 | 6.13 | 925.4 | 78.8 | 1.212738 | 0.045596 | Pumped |
| Mt. Gate | August | 2 | FeOB-rich | Anthracite | 2 | 11.9 | 6.18 | 922.7 | 74.8 | 1.216382 | 0.045145 | Pumped |
| Mt. Gate | August | 3 | FeOB-rich | Anthracite | 2 | 11.9 | 6.18 | 924.3 | 70.4 | 1.211697 | 0.045426 | Pumped |
| Mt. Gate | December | 1 | FeOB-rich | Anthracite | 2 | 11.8 | 6.31 | 939.1 | 77 | 1.92893 | 0.04354 | Pumped |
| Mt. Gate | December | 2 | FeOB-rich | Anthracite | 2 | 11.8 | 6.33 | 943.8 | 69.3 | 1.933094 | 0.045038 | Pumped |
| Mt. Gate | December | 3 | FeOB-rich | Anthracite | 2 | 11.8 | 6.35 | 924 | 74.9 | 1.93934 | 0.043211 | Pumped |
| Six Bells | April | 1 | SOB-rich | Medium volatile | 12 | 18.4 | 7.06 | 2804 | 73.7 | 10.48264 | 0.000784 | Pumped |
| Six Bells | April | 2 | SOB-rich | Medium volatile | 12 | 18.6 | 7 | 2414 | 68.2 | 10.30567 | 0.220594 | Pumped |
| Six Bells | April | 3 | SOB-rich | Medium volatile | 12 | 18.2 | 6.98 | 2395 | 73 | 10.47223 | 0.001304 | Pumped |
| Six Bells | August | 1 | SOB-rich | Medium volatile | 12 | 18.6 | 7.12 | 2757 | 76.9 | 8.603675 | 0.000274 | Pumped |
| Six Bells | August | 2 | SOB-rich | Medium volatile | 12 | 18.5 | 7.13 | 2754 | 73.6 | 8.666134 | 5.01E-05 | Pumped |
| Six Bells | August | 3 | SOB-rich | Medium volatile | 12 | 18.6 | 7.12 | 2756 | 74.3 | 8.707773 | 0 | Pumped |
| Six Bells | December | 1 | SOB-rich | Medium volatile | 12 | 18.5 | 6.8 | 2740 | 60.3 | 10.93026 | 0.000521 | Pumped |
| Six Bells | December | 2 | SOB-rich | Medium volatile | 12 | 18.5 | 6.87 | 2741 | 60.1 | 10.99272 | 0.000232 | Pumped |
| Six Bells | December | 3 | SOB-rich | Medium volatile | 12 | 18.5 | 6.86 | 2741 | 60 | 10.87821 | 2.38E-05 | Pumped |
| Taff Bargoed | April | 1 | FeOB-rich | Low volatile | 11 | 11.9 | 6.94 | 498 | 74.9 | 1.152882 | 0.101044 | Pumped |
| Taff Bargoed | April | 2 | FeOB-rich | Low volatile | 11 | 12 | 7.33 | 499.5 | 54.2 | 1.152882 | 0.101215 | Pumped |
| Taff Bargoed | April | 3 | FeOB-rich | Low volatile | 11 | 12 | 7.39 | 499.7 | 78.5 | 1.169017 | 0.101113 | Pumped |
| Taff Bargoed | August | 1 | FeOB-rich | Low volatile | 11 | 12.3 | 6.88 | 684.4 | 82.2 | 1.141431 | 0.112942 | Pumped |
| Taff Bargoed | August | 2 | FeOB-rich | Low volatile | 11 | 12.2 | 7.04 | 686 | 57.1 | 1.138829 | 0.111375 | Pumped |
| Taff Bargoed | August | 3 | FeOB-rich | Low volatile | 11 | 12.1 | 7.07 | 689 | 45.1 | 1.134144 | 0.109431 | Pumped |
| Taff Bargoed | December | 1 | FeOB-rich | Low volatile | 11 | 12.1 | 6.78 | 508 | 76.5 | 1.40688 | 0.102321 | Pumped |
| Taff Bargoed | December | 2 | FeOB-rich | Low volatile | 11 | 12 | 6.85 | 698 | 39.6 | 1.396991 | 0.100236 | Pumped |
| Taff Bargoed | December | 3 | FeOB-rich | Low volatile | 11 | 12 | 6.86 | 698.8 | 59.2 | 1.383979 | 0.103107 | Pumped |
| Taff's Well | April | 1 | FeOB-rich | N/A | n/a | 20.9 | 7.06 | 505.9 | 26.1 | 0.236094 | 0.028558 | Gravity-driven |
| Taff's Well | April | 2 | FeOB-rich | N/A | n/a | 21 | 7.18 | 506.4 | 18.7 | 0.235989 | 0.029768 | Gravity-driven |
| Taff's Well | April | 3 | FeOB-rich | N/A | n/a | 21.1 | 7.19 | 506.3 | 21.4 | 0.234116 | 0.033626 | Gravity-driven |
| Taff's Well | August | 1 | FeOB-rich | N/A | n/a | 21.3 | 7.23 | 528.4 | 18.7 | 0.233803 | 0.024662 | Gravity-driven |
| Taff's Well | August | 2 | FeOB-rich | N/A | n/a | 21.1 | 7.42 | 511.5 | 22.3 | 0.233387 | 0.024413 | Gravity-driven |
| Taff's Well | August | 3 | FeOB-rich | N/A | n/a | 21.2 | 7.35 | 511.8 | 19 | 0.234116 | 0.024649 | Gravity-driven |
| Taff's Well | December | 1 | FeOB-rich | N/A | n/a | 21.2 | 7.29 | 515.3 | 24 | 0.230056 | 0.024726 | Gravity-driven |
| Taff's Well | December | 2 | FeOB-rich | N/A | n/a | 21.2 | 7.29 | 516.7 | 33 | 0.229952 | 0.02481 | Gravity-driven |
| Taff's Well | December | 3 | FeOB-rich | N/A | n/a | 21 | 7.29 | 519.5 | 38.7 | 0.229327 | 0.024433 | Gravity-driven |
| Ynysarwed | April | 1 | FeOB-rich | Low volatile | 6 | 13.2 | 6.12 | 1558 | 35.3 | 8.280972 | 1.401179 | Gravity-driven |
| Ynysarwed | April | 2 | FeOB-rich | Low volatile | 6 | 13 | 6.19 | 1568 | 35.6 | 8.442324 | 1.400485 | Gravity-driven |
| Ynysarwed | April | 3 | FeOB-rich | Low volatile | 6 | 13 | 6.14 | 1573 | 35 | 8.541216 | 1.392531 | Gravity-driven |
| Ynysarwed | August | 1 | FeOB-rich | Low volatile | 6 | 13 | 6.18 | 1602 | 31.4 | 8.525602 | 1.454526 | Gravity-driven |
| Ynysarwed | August | 2 | FeOB-rich | Low volatile | 6 | 13 | 6.31 | 1601 | 31.3 | 8.556831 | 1.431995 | Gravity-driven |
| Ynysarwed | August | 3 | FeOB-rich | Low volatile | 6 | 13 | 6.33 | 1604 | 31.1 | 8.744207 | 1.447091 | Gravity-driven |
| Ynysarwed | December | 1 | FeOB-rich | Low volatile | 6 | 12.9 | 6.26 | 1633 | 32.8 | 11.34665 | 1.551977 | Gravity-driven |
| Ynysarwed | December | 2 | FeOB-rich | Low volatile | 6 | 12.9 | 6.35 | 1633 | 32.8 | 11.58607 | 1.540401 | Gravity-driven |
| Ynysarwed | December | 3 | FeOB-rich | Low volatile | 6 | 12.9 | 6.35 | 1632 | 32.6 | 12.03369 | 1.472895 | Gravity-driven |

**Table S2 –** Summarised geochemistry data per site and month. Values are given as mean ± 1 standard deviation (n=3).


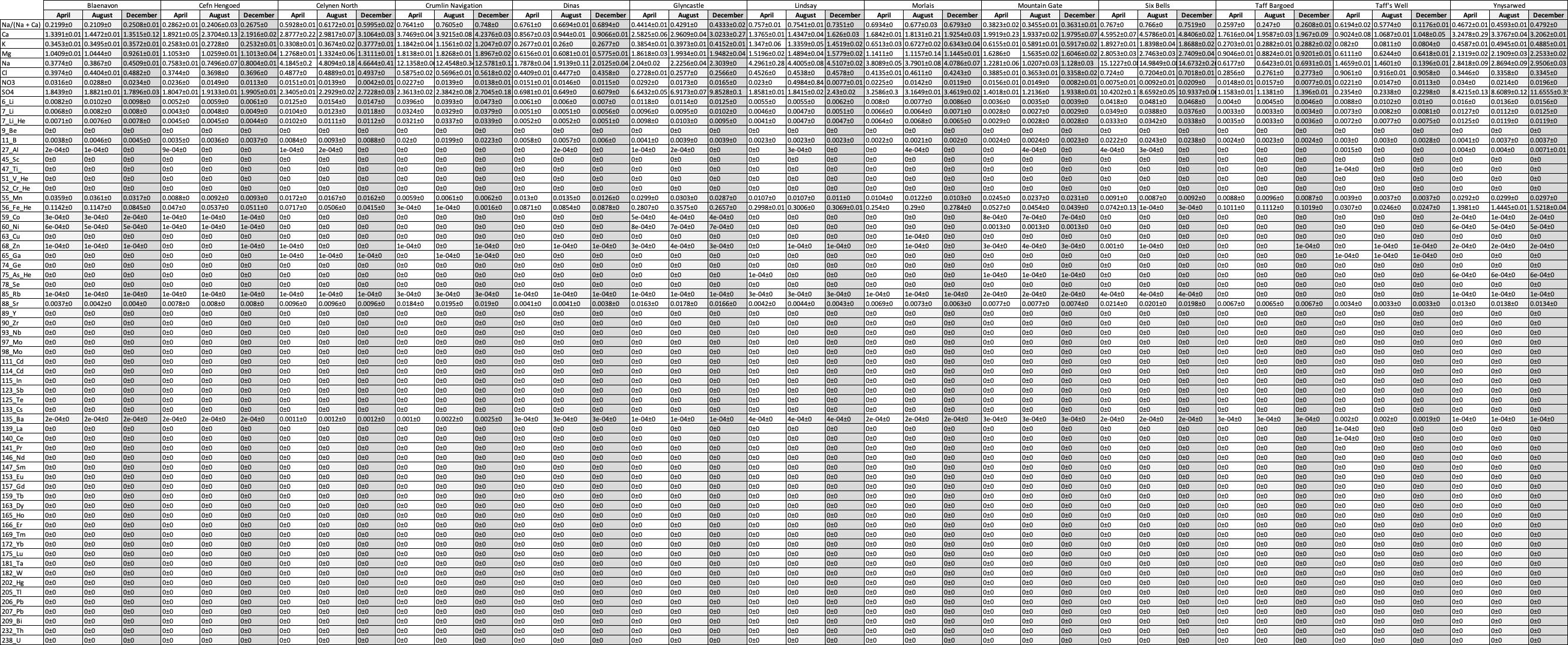

Supplement: fiaf039_Supplemental_File [file fiaf039_supplemental_file.docx]
